# Monitoring Electric Impedance During Freezing and Thawing of Saline and De-ionized Water

**DOI:** 10.2478/joeb-2020-0016

**Published:** 2020-12-31

**Authors:** Sisay Mebre Abie, Daniel Münch, Joakim Bergli

**Affiliations:** 1 Department of Physics, University of Oslo, 0316 Oslo, Norway; 2 Faculty of Ecology and Natural Resource Management, Norwegian University of Life Sciences, 1432 Aas, Norway; 3 Animalia – Norwegian Meat and Poultry Research Centre, 0585 Oslo, Norway

**Keywords:** electrical impedance, freezing, thawing, solidification, ion mobility, electron transfer

## Abstract

Physiological saline (0.9% NaCl) and deionized water were frozen in a laboratory chest freezer and impedance was monitored throughout freezing and thawing. The resistive and reactive components of electrical impedance were measured for these samples during freezing and thawing (heating) within a temperature range between 20 °C and −48 °C. The impedance of saline solution and de-ionized water increases sharply at the freezing point, similar to what is known for, e.g., complex tissues, including meat. Yet, only the saline solution impedance shows another sharp increment at a temperature between −30 and −20 °C. Changes of the electric properties after solidification suggest that the latter is linked to transformations of the ice lattice structure. We conclude that the electrical properties might serve as sensitive indicators of these phase changes.

## Introduction

Ice is one of the three phases of water that plays a very important role in the environment and human life. In the 1950s to 1980s much research had been dedicated to advancing the understanding of the low frequency properties of ice and snow, and to exploiting these properties for glaciological purposes [1,2,3]. The electrical properties are very sensitive to the purity of the crystal and the properties of the material itself. An understanding of these properties is essential in fields such as glaciology, ice mechanics, meteorology, thunderstorm electricity, and more. Many researchers have been investigating the dielectric properties of the natural occurring ice, such as snow, sea ice, glacial ice and lake ice [4,5,6,7,8]. According to their investigation, such natural occurring ice always contains many impurities and the effects of such impurities determine the electrical properties. Other authors also studied the dielectric properties of pure ice from a viewpoint of molecular structure of ice and found that pure ice has a relaxation spectrum [5, 9,10,11], which can be characterized by the single-dispersion Debye relaxation phenomenon that is described by the Debye dispersion equation,

$$Z=R_{\infty }+\frac{R_{0}-R_{\infty }}{1+j\omega \tau }$$

where R_0_ is the resistance at very low frequency (static value), *R*_∞_ the resistance at very high frequency, ω is angular frequency and τ is the relaxation time.

Jaccard [12] and Petrenko [13] describe the electric conductivity of ice. According to their report, the electrical properties of ice can be explained by the presence of four mobile structural defects of the lattice, which are the ions H_3_O^+^ and OH^−^, and the Bjerrum or valence defects. Petrenko [13] reported later a similar result. When an external electric field is applied to ice, protonic defects move to cancel this field [3, 12]. Ionic defects move by sliding hydrogen (H) to the other side of a hydrogen bond. Bjerrum defects move by jumping an H from its current hydrogen bond to another. Since the latter move takes more energy, Bjerrum defects are more temperature dependent than ionic defects [3, 14].

Furthermore, the electrical properties of ice are very sensitive to small concentrations of certain impurities that can be incorporated in the hydrogen-bonded network to generate protonic point defects [3]. Many researchers have investigated the electric properties of pure and impure ice (artificial sea ice that contain different amount of salt concentration), either in the lab or outdoors with field tests [6, 8, 15,16,17]. Impedance spectroscopy methods were used in some studies to understand the nature of ice. Chin [18] also used AC impedance to measure the impedance of various concentrations of KCl, KOH, MgSO4, and HCl. Impedance spectroscopy, therefore, is a promising approach to the study of these phenomena.

In this paper, we use impedance spectroscopy to measure the impedance of different types of ice in the laboratory. The goal of this experiment is to gain insight into the electrical properties of the physiological saline and deionized water during the freezing and melting process. Physiological salt solution (0.9% NaCl solution) resembles key features of intra- and extracellular fluids of biological materials, such as muscle tissue. Impedance measurements are of a high interest because they can provide relevant information for meat characterization. Meat is an aggregation of cells in about 60–80 % of fluid. Two thirds of fresh meat fluid is inside the cell and one third is in the extracellular space. Both intracellular and extracellular fluids are electrolytes containing free ions able to transport electrical charge. However, not much is known about impedance in frozen meat and the electrolytes it contains.

Electrical impedance will be used to identify factors that influence the ice formation of saline solution and de-ionized water and that make impact on either the quality of frozen biological materials or their electrical properties. Moreover, this experiment will allow to link electrical impedance measurements of ice with freezing rate and temperature.

## Materials and methods

In these fundamental laboratory studies on ice crystals, the electric impedance of ice was measured using a Zürich Instruments MFIA impedance analyzer. The frequency range was from 10 Hz to 1 MHz and the applied voltage was 600 mV rms. A tetrapolar electrode setup was used. The stainless steel needle electrodes had diameters of 2 mm and a length of 12 mm with 18 mm distance between the middle pick-up electrodes. Electrode pins were inserted into the upper surface of the tested solutions. 0.9% physiological saline solution and deionized water was used as samples for impedance measurements as a function of temperature.

The sample holder was made of a plastic cup. Freezing was carried out in a commercial chest freezer (TM 400 Frigor, Vibocold A/S, Denmark) that was set to a temperature of −50±2 °C. The internal dimensions of the freezing chamber were 116 × 60 × 85 cm^3^ (width × depth × height). The sample, as well as the freezer chamber temperature, was measured using calibrated T-type thermocouples, which were connected to Lutron TM-947SD four-channel thermometer (Lutron Electronic, Taiwan). Temperature at the center of the sample was obtained by inserting a T-type thermocouple at a pre-determined location. Surrounding air temperature was measured close to the cup. The measurement was carried out continuously under freezing before and after solidification and under subsequent heating.

### Methods according to protocol

We grew a 500 ml sample of physiological saline ice and monitored its impedance throughout its growth. We prepared experiments based on the following procedure:

1. The chest freezer was set to the required temperature 12 hours before the experiment.
2. The impedance analyser was placed close to the chest freezer for subsequent measurements.
3. Sample and electrode – connected with the impedance analyser cables – were moved into the freezer.
4. The temperature sensors were placed inside and outside the sample (in the freezing chamber). The temperatures was recorded in 1 second intervals.
5. Then the impedance was measured in defined temperature intervals until the sample's centre temperature was close to the freezing chamber temperature. At this point we left the sample in the freezer for 24 hours. To subsequently perform the impedance measurements during thawing (defrosting) samples were transferred to an incubator (20 °C). Impedance was measured until the sample's centre temperature reached 15 °C.

### Ethical approval

The conducted research is not related to either human or animal use.

## Results

**Figure 1:**
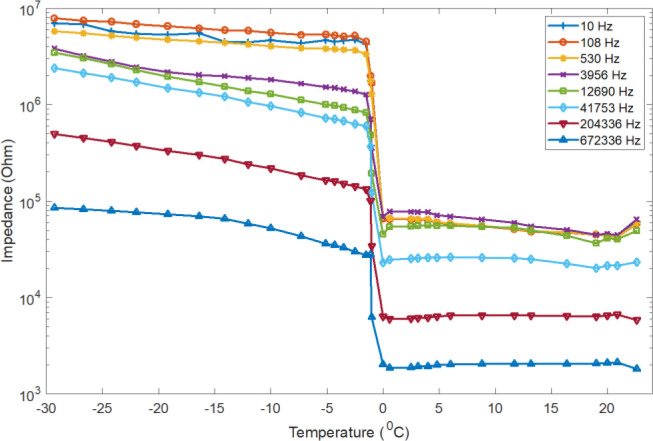
De-ionized water impedance at selected frequencies as a function of temperature measured during freezing.

The impedance of de-ionized water was measured during freezing as well as heating (melting). The impedance measurements were conducted in dynamical conditions of temperature and phase transformation, so the curves can be a little distorted. A typical temperature dependence of impedance measurement result for different selected frequencies, is shown in figure 1. As the figure demonstrates, the deionized water freezes at 0 °C and the electrical impedance for liquid deionized water increase as the temperature decrease from 20 to 0 °C. The electrical impedance also keeps increasing as the temperature drops below the freezing point. However, in the heating (melting) process the electrical impedance decreases as a function of temperature from −48 °C to the melting point temperature as demonstrated in the figure 3.

**Figure 2:**
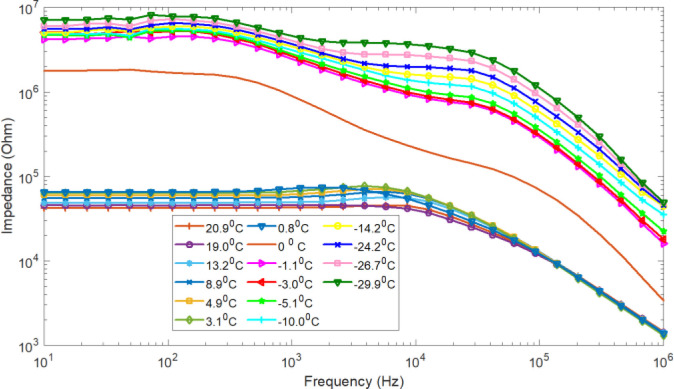
De-ionized water impedance as a function of frequency for different temperatures measured during freezing.

**Figure 3:**
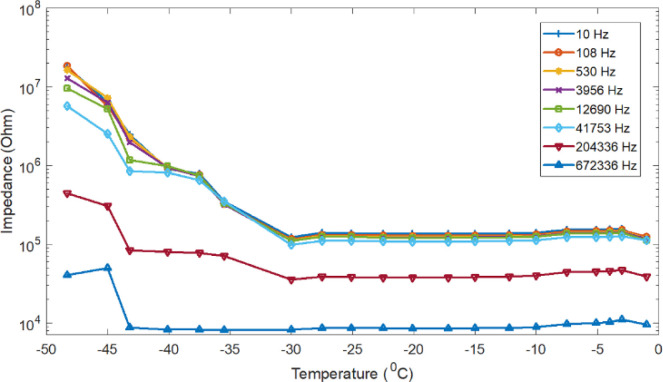
De-ionized water ice impedance of six selected frequencies as a function of temperature measured during heating (thawing).

**Figure 4:**
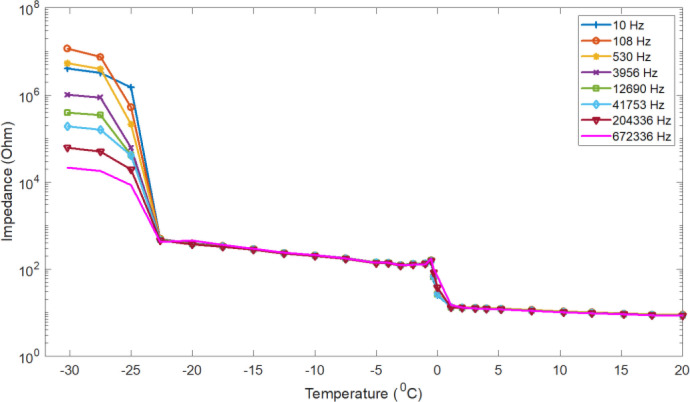
Physiological saline (0.9% NaCl salt solution) impedance of selected frequencies as a function of temperature measured during freezing.

Similarly, typical temperature dependency curves for electrical impedance data of selected frequencies for 0.9% physiologic saline from −30 to 20 °C, are shown in Figure 4.

As the figure demonstrates, the saline electrical impedance increases with temperature decrement. Starting from 20 °C, the impedance increase very slowly as the temperature falls down to the freezing point.

When the temperature falls below the freezing points, the impedance keeps increasing. This result is in contradiction with the result reported by Sauerheber [19], which says that the conductivity of 0.9% NaCl saline solution increases as temperature falls further below the freezing temperature.

In two different temperature ranges, between −2 and 2 °C, and from −30 to −20 °C, the impedance increases sharply.

**Figure 5:**
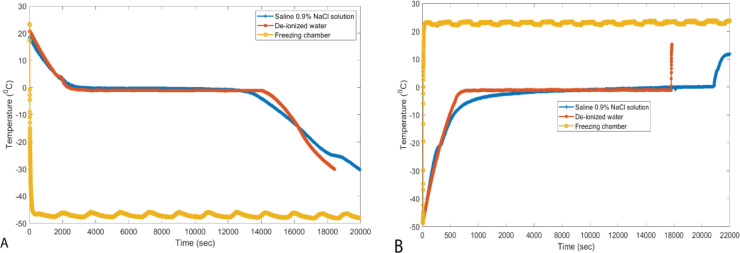
Typical freezing curves of 500 ml saline and de-ionized water. A) during freezing and B) during melting.

**Figure 6:**
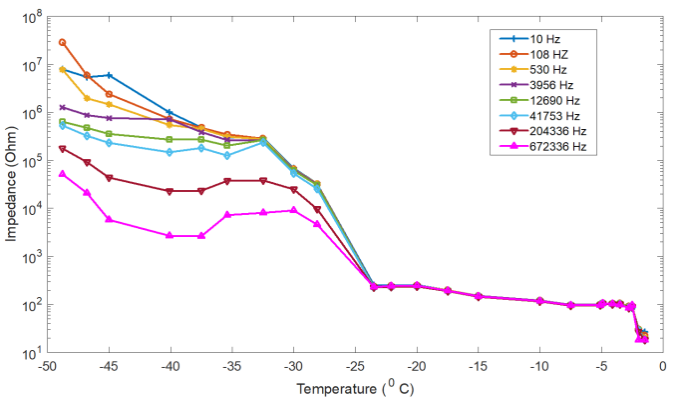
Physiological saline (0.9% NaCL salt solution) ice impedance of selected frequencies as a function of temperature measured during heating (unfrozen process)

Figure 5 shows the typical temperature changes that occur in the physiological saline and de-ionized water freezing and heating process. The curve for the samples shows two regions were the temperature is close to time independent, the first one is the long interval at the freezing points (0 °C) in which most of the thermal arrest (latent heat release) occurs and the second one is a small region that occurs between a temperature of −30 °C and −20 °C. These two regions correspond to the sharp impedance increment that is shown in figure 4. Similarly, the heating (melting) temperature curve of saline has two regions were the temperature is close to time independent as shown in the figure 5B, the first one is the small interval that occurs between the temperatures −30 °C and −20 °C and the second is the big interval at the melting points (0 °C). In both cases, most of the energy goes exclusively to changing the phase of the substance; it does not go into changing the temperature of the substance. These two phenomena correspond to the sharp decrement of the impedance as shown figure 6.

The physiologic saline electric impedance was also measured during heating (melting) from −48 to 0 °C. Figure 6 presents data showing the electrical impedance of saline ice as a function of temperature. The electric impedance decreases linearly from −48 to −30 °C and decreases very sharply in the temperature range from −30 to −20 °C and around the melting point, and then from −20 °C to the melting temperature, it shows a very slow decrement. This behaviour is similar to the freezing process of the same materials as shown above.

## Discussion

The motion or the kinetic energy of molecules and ions directly depends on temperature. Different composition, therefore, explains that temperature effects on electrical properties can differ among materials. With rising temperature, inter-ionic attraction decreases resulting in reduced viscosity and increased mobility (ions of the materials get more energy to vibrate and move around). In addition to this, with temperature increment the degree of dissociation of electrolyte increases, hence the number of ions also increases for conduction [20]. At low temperature, in contrast, the molecules or ions are losing energy and become dormant or bounded in a specific location and structure. Therefore, there is no free electron in pure ice at a temperature below the freezing point, the charge carriers are the proton jumps. Based on Jaccard [21] and Petrenko [13], the electrical properties of ice can be explained by the presence of four mobile structural defects of the lattice, which are the ions H_3_O^+^ and OH^−^, and the Bjerrum or valence defects.

If the ice contains any impurities, then the electrical properties largely depend on those impurities [8]. Thus, the DC conductivity is determined by the opposite action of both sorts on the proton configuration. The existence of the high amount of one of them is necessary for the occurrence of the high dielectric constant and its Debye relaxation. The difference of the impedance measurement results of the ice of 0.9% NaCl saline solutions and de-ionized water indicate that the salt ions play an important role in the electrical conduction of the ice crystal lattice.

In the last 50 years, the suggestion of electrical point defects in ice, i.e. Bjerrum L (bond with no hydrogens on it), D (bond with two hydrogens) defects, and ionic defects, shaped our understanding of the dielectric relaxation and electrical conductivity of ice. Bjerrum defect states (DL) have mobilities in the same order of magnitude as that generally occurring for ionic transport. This suggests that thermal activation over a potential threshold facilitates the transfer of protons through the jumping of Bjerrum defect states to a neighbouring bond. Therefore, in the ice phase, the impedance of the results can be characterized by the single dispersion Debye relaxation phenomenon.

A method to characterize the Debye relaxation is using the Debye relaxation time constant [9], which is highly sensitive to temperature and described by the equation

$$\tau =A\cdot \exp\left(\frac{E}{kT}\right)$$

Here, the *A* is a coefficient, *E* is the activation energy barrier of the dielectric relaxation (eV), *k* is the Boltzmann constant (8.618 · 10^−5^ eV/K), and *T* is the absolute temperature (K).

The frequency and temperature dependences of pure ice (ice of de-ionized water) that we observed here were very similar to the results obtained by other authors. In the impedance, for de-ionized water, a dispersion is only found for the high frequency range but, in the low frequency range, such a dispersion is not observed. However, both low frequency and high frequency dispersions were observed in the impedance of the ice of the de-ionized water. In the liquid physiological saline solution impedance, no dispersion was observed. Nevertheless, the dispersion started to appear in the ice state at lower temperature. Figure 2 shows, in accordance with interpretation presented in the literature (Rusiniak [11]), that the double dispersion in pure ice arises from one dispersion in de-ionized water that is split during solidification.

Both the frozen solid salt water and de-ionized water behave like a semiconductor where electrons enter conduction bands as temperature increases, the charge carriers (protons) between a ‘valence’ band (H-bonded H_2_O) and a ‘conduction’ band (excess protons fluctuating in H bonds), which exhibit increasing impedance with decreasing temperature [22].

Here we are not comparing the behaviour of protons in hydrogen-bonded media with that of charge carriers in electronic semi-conductors that has already been performed by many research groups for the last half century. However, the way in which the electric impedance result compare to the electric impedance of frozen meat is also our interest. The impedance of the meat [23] changes with a different rate than the impedance of the pure ice as well as the saline ice. The difference in electrical impedance of pure ice, saline ice and frozen meat strongly suggests that the impurities play a dominant role in the lattice mobility of the ice. Nevertheless, all the three different materials have impedance, which increases as the temperature decreases.

Furthermore, the impedance of meat, saline solution and de-ionized water increases sharply at the freezing point, but the saline solution impedance shows another sharp increment at a temperature between −30 and −20 °C. These increments can be explained, as temperature is a measure of “average kinetic energy”, any change in temperature is a change in kinetic energy. As shown in figure 5, there are two regions were the temperature is close to time independent around freezing points and at a temperature between −30 and −20 °C. Within these regions that correspond to sharp impedance increments, there is no change in temperature, so kinetic energy remains constant. However, all the energy that is absorbed or released is related to changes in potential energy. As the sample freeze, after nucleation, the crystal formation (i.e. the change of liquid water to solid ice) happens at a constant temperature accompanied by latent heat removal. During this process, the ions or molecules become bounded into the crystal.

However, saline ice is more complex than pure ice. During freezing, the ice lattice as it forms, rejects the salts, which remain in the liquid brine entrapped within crystalline zones (or “grains”) formed from platelets of pure ice separated by ordered rows of brine-filled pockets or cells. Brine is also located between the grains. This brine remains unfrozen until the temperature reaches below −20 °C and then start the crystallization. This implies that the presence of this brine plays an important role in shaping the behaviour of the substance. In fact, according to Assur [24], the temperature and salinity dependence of the volume of brine, is one of the principal contributing factors to the temperature variation of any physical property.

In accordance with interpretation presented in the literature [11], during solidification the dipole moment of water molecule decreases [25], and, as a result will lead to a decrease in density of positive surface charge. This may lead to the sharp increase of the impedance of all the samples during the solidification process. Changes of the electric properties after solidification suggest a transformation of lattice structure. Therefore, the electrical properties might serve as sensitive indicators of these phase changes, as well as of the final solidus point for the material. It has turned out that an interesting additional feature arises from the proton relaxation mechanism. Furthermore, those two sharp changes of impedance can be attributed to the transition of major charge carriers in the sample from ionic defect to rotational or Bjerrum defects.

## Conclusion

It is apparent from our limited observations, that the electrical properties of physiological saline ice and deionized ice are appreciably different from those of meat ice (frozen meat). The greatest difference appears in the impedance change rate (increasing and decreasing rate as a function of temperature). In all the three materials, impedance increases sharply during initial ice crystallization. Temperature and ice crystal structure appear to play a more important role in controlling the electrical parameters. In addition, at higher frequencies the effect of temperature is considerably less. We have also shown that pure ice has two dispersion spectrum related to temperature. Saline ice has two different temperature points where crystallisation occurs.

## References

[1] Buchanan S, Ingham M, Gouws G (2011). The low frequency electrical properties of sea ice. Journal of Applied Physics.

[2] Kulessa B (2007). A critical review of the low-frequency electrical properties of ice sheets and glaciers. Journal of Environmental and Engineering Geophysics.

[3] Victor F (2010). Petrenko and Robert W. Whitworth. Physics of Ice.

[4] Cumming WA (1952). The dielectric properties of ice and snow at 3.2 centimeters. Journal of Applied Physics.

[5] Evans S (1965). Dielectric properties of ice and snow-a review. Journal of Glaciology.

[6] Fujino K (1967). Electrical properties of sea ice. Physics of Snow and Ice: proceedings.

[7] Kuroiwa D (1954). The dielectric property of snow.

[8] Levi L (1967). Electrical properties of ice doped with different electrolytes. Physics of Snow and Ice: proceedings.

[9] Auty RP, Cole RH (1952). Dielectric properties of ice and solid D2O. The Journal of Chemical Physics.

[10] Murphy EJ (1934). The temperature dependence of the relaxation time of polarizations in ice. Transactions of the Electrochemical Society.

[11] Rusiniak L (2004). Electric properties of ice near solidification and melting temperature. Acta Geophysica Polonica.

[12] Jaccard C (1965). Mechanism of the electrical conductivity in ice. Annals of the New York Academy of Sciences.

[13] Petrenko VF (1993). Electrical properties of ice.

[14] Stillman DE, Grimm RE (2008). Electrical properties of ice and implications for solar system exploration. LPI.

[15] Addison JR (1970). Electrical relaxation in saline ice. Journal of Applied physics.

[16] Addison J.R (1975). Electrical properties of saline ice at 1 kHz down to −150° C. Journal of Applied Physics.

[17] Wentworth F.L., Cohn M. (1964). Electrical properties of sea ice at 0.1 to 30 Mc/s. J. Res. NBS.

[18] Chin KB, Buehler MG, Seshadri S, Keymeulen D, Anderson RC, Dutz S, Narayanan SR (2007). Investigation of water and ice by ac impedance using electrochemical properties cup. Review of Scientific Instruments.

[19] Sauerheber R, Heinz B (2015). Temperature effects on conductivity of seawater and physiologic saline, Mechanism and Significance. Chem. Sci. J..

[20] Barron JJ, Ashton C (2005). The effect of temperature on conductivity measurement. TSP.

[21] Jaccard C (1959). Theoretical and experimental studies of the electrical properties of ice. Helv. Phys. Acta.

[22] Eigen M, De Maeyer L (1958). Self-dissociation and protonic charge transport in water and. Proceedings of the Royal Society of London. Series A. Mathematical and Physical Sciences.

[23] Abie SM, Martinsen ØG, Egelandsdal B, Hou J, Bjerke F, Mason A, Münch D Feasibility of using electrical impedance sensing for assessment of the damage of biological cells during freezing and thawing.

[24] Assur A (1960). Composition of sea ice and its tensile strength.

[25] Owston PG (1958). The structure of ice-I, as determined by x-ray and neutron diffraction analysis. Advances in Physics.

